# Isopropyl 3-(3,4-dihydroxy­phen­yl)-2-hydroxy­propanoate

**DOI:** 10.1107/S1600536810013334

**Published:** 2010-04-17

**Authors:** Ye-Fei Nan, Qun-Zheng Zhang, Xu-Ji Shen, Xin-Feng Zhao, Xiao-Hui Zheng

**Affiliations:** a College of Life Scineces, Northwest University, Xi’an 710069, People’s Republic of China; b College of Chemistry & Chemical Engineering, Xi’an Shiyou University, Xi’an 710065, People’s Republic of China

## Abstract

The title compound, C_12_H_16_O_5_, is a derivative of β-(3,4-dihydroxy­phen­yl)-α-hydr­oxy acid. The crystal packing is stabilized by inter­molecular O—H⋯O hydrogen bonds.

## Related literature

For the anti­oxidant properties and vasorelaxant activity of the title compound, see: Tian *et al.* (2008 ▶); Wang *et al.* (2008 ▶). For the preparation, see: Zhang *et al.* (2009 ▶).
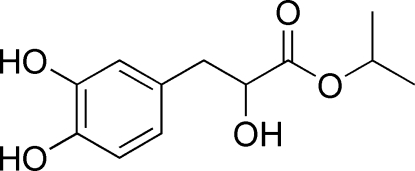


## Experimental

### 

#### Crystal data

C_12_H_16_O_5_
*M* *_r_* = 240.25
Monoclinic, 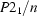

*a* = 5.7691 (13) Å
*b* = 14.271 (3) Å
*c* = 14.955 (3) Å
β = 96.360 (3)°
*V* = 1223.7 (5) Å^3^
*Z* = 4
Mo *K*α radiation
μ = 0.10 mm^−1^
*T* = 296 K
0.38 × 0.27 × 0.18 mm

#### Data collection

Bruker SMART CCD area-detector diffractometer
5934 measured reflections
2174 independent reflections
1598 reflections with *I* > 2σ(*I*)
*R*_int_ = 0.029

#### Refinement

*R*[*F* ^2^ > 2σ(*F* ^2^)] = 0.038
*wR*(*F* ^2^) = 0.106
*S* = 1.14
2174 reflections
159 parameters
H-atom parameters constrained
Δρ_max_ = 0.18 e Å^−3^
Δρ_min_ = −0.18 e Å^−3^

### 

Data collection: *SMART* (Bruker, 1997 ▶); cell refinement: *SAINT* (Bruker, 1997 ▶); data reduction: *SAINT*; program(s) used to solve structure: *SHELXS97* (Sheldrick, 2008 ▶); program(s) used to refine structure: *SHELXL97* (Sheldrick, 2008 ▶); molecular graphics: *SHELXTL* (Sheldrick, 2008 ▶); software used to prepare material for publication: *SHELXTL*.

## Supplementary Material

Crystal structure: contains datablocks I, global. DOI: 10.1107/S1600536810013334/jh2138sup1.cif

Structure factors: contains datablocks I. DOI: 10.1107/S1600536810013334/jh2138Isup2.hkl

Additional supplementary materials:  crystallographic information; 3D view; checkCIF report

## Figures and Tables

**Table 1:** Hydrogen-bond geometry (Å, °)

*D*—H⋯*A*	*D*—H	H⋯*A*	*D*⋯*A*	*D*—H⋯*A*
O1—H1⋯O4^i^	0.82	1.96	2.7621 (14)	164
O2—H2⋯O3^ii^	0.82	1.93	2.7417 (15)	169
O3—H3⋯O1^iii^	0.82	2.00	2.7832 (14)	160

Symmetry codes: (i) 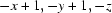; (ii) 
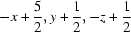
; (iii) 
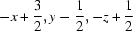
.

**Fig. 1.**
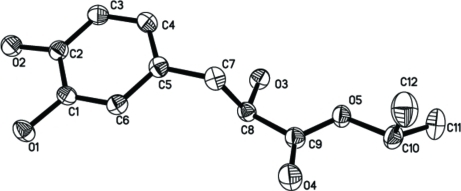
The molecular structure of (I), with atom labels and 30% probability displacement ellipsoids for non-H atoms.

**Fig. 2.**
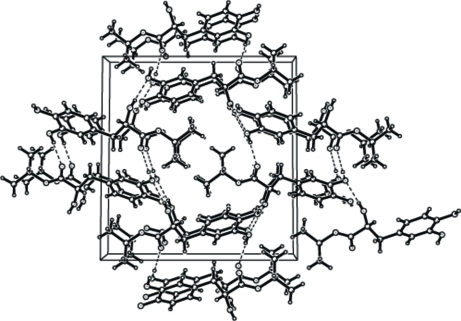
The packing of (I), viewed down the a axis, molecules are connected by O—H···O hydrogen bonds shown as dashed lines.

## supplementary crystallographic information

### Comment

The antioxidant property (Tian *et al.*, 2008) and  vasorelaxant activity (Wang *et al.*, 2008) of the title compound (I) is already described. At 296 K, X-ray structure analysis was carried out in order to structurally characterised (I). The molecular structure of the title compound and the atom-numbering scheme are shown in Fig.1. In the Fig.1, the hydrogen atoms are omitted for clarity. As shown in Fig.2, both the carbonyl oxygen and the hydroxyl oxygen form hydrogen bonds with the hydrogen of the hydroxyl in another moleculars. The distance of O1 and O4 is 2.7622 (14)/%A. The distance is longer than that between O2 and O3(2.7417/%A), shorter than O3 and O1(2.7832/%A). All the data, listed in table 1 suggest strong hydrogen bond interactions.

### Experimental

The synthesis of the crude product was carried out according to reported methods(Zhang *et al.*, 2009). The title compound was crystalised from ether and water at room temperature. Spectroscopic analysis: IR(KBr, χm^-1^): 3256, 2952, 1678, 1656; ^1^H NMR (DMSO, δ, p.p.m.): 12.389 (s, 1 H), 9.492 (s, 1 H), 9.316 (s, 1 H), 7.286—7.282 (d, 1 H), 7.196 (s, 1 H), 7.088—7.068 (m, 1 H), 6.797—6.781 (d, 1 H), 3.771 (s, 3 H), 1.985 (s, 3 H).

### Refinement

H atoms bonded to N and O atoms were located in a difference map and refined with distance restraints of O—H = 0.8200 and N—H = 0.8600 Å, and with *U*ĩso~(H) = 1.2*U*~eq~(N,*O*). Other H atoms were positioned geometrically and refined using a riding model (including free rotation about the ethanol C—C bond), with C—H = 0.93–0.96 Å.

### Crystal data

**Table:**

C_12_H_16_O_5_	*D*_x_ = 1.304 Mg m^−^^3^
*M**_r_* = 240.25	Melting point: 360 K
Monoclinic, *P*2_1_/*n*	Mo *K*α radiation, λ = 0.71073 Å
*a* = 5.7691 (13) Å	Cell parameters from 2102 reflections
*b* = 14.271 (3) Å	θ = 2.7–25.9°
*c* = 14.955 (3) Å	µ = 0.10 mm^−^^1^
β = 96.360 (3)°	*T* = 296 K
*V* = 1223.7 (5) Å^3^	Block, colorless
*Z* = 4	0.38 × 0.27 × 0.18 mm
*F*(000) = 512	

### Data collection

**Table:**

Bruker SMART CCD area-detector diffractometer	1598 reflections with *I* > 2σ(*I*)
Radiation source: fine-focus sealed tube	*R*_int_ = 0.029
graphite	θ_max_ = 25.1°, θ_min_ = 2.0°
phi and ω scans	*h* = −6→6
5934 measured reflections	*k* = −17→9
2174 independent reflections	*l* = −17→17

### Refinement

**Table:**

Refinement on *F*^2^	Primary atom site location: structure-invariant direct methods
Least-squares matrix: full	Secondary atom site location: difference Fourier map
*R*[*F*^2^ > 2σ(*F*^2^)] = 0.038	Hydrogen site location: inferred from neighbouring sites
*wR*(*F*^2^) = 0.106	H-atom parameters constrained
*S* = 1.14	*w* = 1/[σ^2^(*F*_o_^2^) + (0.0576*P*)^2^] where *P* = (*F*_o_^2^ + 2*F*_c_^2^)/3
2174 reflections	(Δ/σ)_max_ < 0.001
159 parameters	Δρ_max_ = 0.18 e Å^−^^3^
0 restraints	Δρ_min_ = −0.18 e Å^−^^3^

### Special details

**Table:**

Geometry. All esds (except the esd in the dihedral angle between two l.s. planes) are estimated using the full covariance matrix. The cell esds are taken into account individually in the estimation of esds in distances, angles and torsion angles; correlations between esds in cell parameters are only used when they are defined by crystal symmetry. An approximate (isotropic) treatment of cell esds is used for estimating esds involving l.s. planes.
Refinement. Refinement of *F*^2^ against ALL reflections. The weighted *R*-factor wR and goodness of fit *S* are based on *F*^2^, conventional *R*-factors *R* are based on *F*, with *F* set to zero for negative *F*^2^. The threshold expression of *F*^2^ > σ(*F*^2^) is used only for calculating *R*-factors(gt) etc. and is not relevant to the choice of reflections for refinement. *R*-factors based on *F*^2^ are statistically about twice as large as those based on *F*, and *R*- factors based on ALL data will be even larger.

### Fractional atomic coordinates and isotropic or equivalent isotropic displacement parameters (Å^2^)

**Table:**

	*x*	*y*	*z*	*U*_iso_*/*U*_eq_	
O1	0.71641 (18)	0.75521 (7)	0.11545 (6)	0.0508 (3)	
H1	0.6220	0.7390	0.0733	0.076*	
O2	1.09466 (18)	0.79570 (7)	0.22404 (8)	0.0554 (3)	
H2	1.2317	0.8059	0.2423	0.083*	
O3	0.94395 (17)	0.34163 (7)	0.23630 (6)	0.0457 (3)	
H3	0.8648	0.3203	0.2738	0.068*	
O4	0.5296 (2)	0.29738 (8)	0.04573 (8)	0.0709 (4)	
O5	0.77917 (17)	0.20274 (7)	0.12753 (6)	0.0464 (3)	
C1	0.8694 (2)	0.68321 (10)	0.13822 (9)	0.0384 (4)	
C2	1.0709 (2)	0.70525 (10)	0.19423 (10)	0.0427 (4)	
C3	1.2329 (3)	0.63583 (11)	0.21636 (11)	0.0535 (4)	
H3A	1.3698	0.6499	0.2527	0.064*	
C4	1.1939 (3)	0.54492 (11)	0.18498 (11)	0.0518 (4)	
H4	1.3058	0.4991	0.2004	0.062*	
C5	0.9928 (2)	0.52155 (10)	0.13152 (9)	0.0411 (4)	
C6	0.8304 (3)	0.59214 (10)	0.10876 (9)	0.0405 (4)	
H6	0.6928	0.5777	0.0730	0.049*	
C7	0.9418 (3)	0.42345 (10)	0.09809 (9)	0.0461 (4)	
H7A	1.0881	0.3911	0.0939	0.055*	
H7B	0.8589	0.4265	0.0381	0.055*	
C8	0.7973 (2)	0.36720 (10)	0.15824 (9)	0.0395 (4)	
H8	0.6727	0.4075	0.1760	0.047*	
C9	0.6870 (3)	0.28507 (11)	0.10523 (10)	0.0439 (4)	
C10	0.6941 (3)	0.12093 (10)	0.07388 (10)	0.0504 (4)	
H10	0.5254	0.1266	0.0577	0.061*	
C11	0.7441 (4)	0.03703 (11)	0.13364 (12)	0.0705 (6)	
H11A	0.6708	0.0450	0.1877	0.106*	
H11B	0.9096	0.0308	0.1486	0.106*	
H11C	0.6840	−0.0183	0.1027	0.106*	
C12	0.8141 (4)	0.11835 (14)	−0.01037 (13)	0.0812 (6)	
H12A	0.9798	0.1149	0.0054	0.122*	
H12B	0.7768	0.1741	−0.0449	0.122*	
H12C	0.7625	0.0644	−0.0454	0.122*	

### Atomic displacement parameters (Å^2^)

**Table:**

	*U*^11^	*U*^22^	*U*^33^	*U*^12^	*U*^13^	*U*^23^
O1	0.0510 (7)	0.0484 (7)	0.0490 (6)	0.0097 (5)	−0.0127 (5)	−0.0090 (5)
O2	0.0500 (7)	0.0482 (7)	0.0644 (8)	−0.0019 (5)	−0.0092 (6)	−0.0115 (5)
O3	0.0503 (6)	0.0477 (7)	0.0368 (5)	−0.0044 (5)	−0.0051 (5)	0.0048 (4)
O4	0.0797 (9)	0.0563 (8)	0.0668 (8)	0.0092 (6)	−0.0370 (7)	−0.0063 (6)
O5	0.0533 (6)	0.0363 (6)	0.0468 (6)	0.0017 (5)	−0.0070 (5)	−0.0023 (4)
C1	0.0381 (8)	0.0432 (9)	0.0330 (7)	0.0041 (6)	0.0004 (6)	0.0008 (6)
C2	0.0434 (9)	0.0430 (10)	0.0411 (8)	−0.0032 (7)	0.0023 (7)	−0.0023 (6)
C3	0.0421 (9)	0.0515 (11)	0.0631 (10)	−0.0005 (8)	−0.0113 (7)	0.0021 (8)
C4	0.0460 (9)	0.0482 (10)	0.0595 (10)	0.0067 (8)	−0.0022 (8)	0.0076 (8)
C5	0.0464 (9)	0.0416 (9)	0.0360 (7)	0.0005 (7)	0.0076 (7)	0.0052 (6)
C6	0.0415 (8)	0.0470 (9)	0.0321 (7)	−0.0023 (7)	−0.0006 (6)	0.0000 (6)
C7	0.0558 (10)	0.0454 (9)	0.0376 (8)	0.0036 (7)	0.0073 (7)	0.0008 (7)
C8	0.0418 (8)	0.0389 (9)	0.0369 (8)	0.0040 (6)	0.0001 (6)	0.0003 (6)
C9	0.0472 (9)	0.0419 (9)	0.0404 (8)	0.0041 (7)	−0.0044 (7)	0.0004 (6)
C10	0.0579 (10)	0.0417 (9)	0.0499 (9)	−0.0054 (7)	−0.0021 (7)	−0.0093 (7)
C11	0.1035 (15)	0.0405 (10)	0.0672 (11)	−0.0069 (10)	0.0076 (11)	−0.0050 (8)
C12	0.1136 (17)	0.0685 (13)	0.0647 (11)	0.0007 (12)	0.0244 (11)	−0.0088 (10)

### Geometric parameters (Å, °)

**Table:**

O1—C1	1.3725 (16)	C5—C7	1.505 (2)
O1—H1	0.8200	C6—H6	0.9300
O2—C2	1.3676 (17)	C7—C8	1.5212 (19)
O2—H2	0.8200	C7—H7A	0.9700
O3—C8	1.4116 (16)	C7—H7B	0.9700
O3—H3	0.8200	C8—C9	1.515 (2)
O4—C9	1.2119 (17)	C8—H8	0.9800
O5—C9	1.3172 (17)	C10—C11	1.503 (2)
O5—C10	1.4698 (17)	C10—C12	1.504 (2)
C1—C6	1.3827 (19)	C10—H10	0.9800
C1—C2	1.392 (2)	C11—H11A	0.9600
C2—C3	1.377 (2)	C11—H11B	0.9600
C3—C4	1.389 (2)	C11—H11C	0.9600
C3—H3A	0.9300	C12—H12A	0.9600
C4—C5	1.375 (2)	C12—H12B	0.9600
C4—H4	0.9300	C12—H12C	0.9600
C5—C6	1.3920 (19)		
			
C1—O1—H1	109.5	O3—C8—C9	114.28 (11)
C2—O2—H2	109.5	O3—C8—C7	107.94 (11)
C8—O3—H3	109.5	C9—C8—C7	108.95 (11)
C9—O5—C10	117.99 (11)	O3—C8—H8	108.5
O1—C1—C6	123.19 (12)	C9—C8—H8	108.5
O1—C1—C2	116.83 (13)	C7—C8—H8	108.5
C6—C1—C2	119.97 (13)	O4—C9—O5	124.32 (14)
O2—C2—C3	124.07 (13)	O4—C9—C8	120.62 (14)
O2—C2—C1	117.15 (13)	O5—C9—C8	115.04 (12)
C3—C2—C1	118.78 (14)	O5—C10—C11	106.12 (12)
C2—C3—C4	120.72 (14)	O5—C10—C12	108.65 (14)
C2—C3—H3A	119.6	C11—C10—C12	113.75 (15)
C4—C3—H3A	119.6	O5—C10—H10	109.4
C5—C4—C3	121.15 (15)	C11—C10—H10	109.4
C5—C4—H4	119.4	C12—C10—H10	109.4
C3—C4—H4	119.4	C10—C11—H11A	109.5
C4—C5—C6	117.93 (14)	C10—C11—H11B	109.5
C4—C5—C7	122.71 (14)	H11A—C11—H11B	109.5
C6—C5—C7	119.37 (13)	C10—C11—H11C	109.5
C1—C6—C5	121.41 (13)	H11A—C11—H11C	109.5
C1—C6—H6	119.3	H11B—C11—H11C	109.5
C5—C6—H6	119.3	C10—C12—H12A	109.5
C5—C7—C8	113.23 (11)	C10—C12—H12B	109.5
C5—C7—H7A	108.9	H12A—C12—H12B	109.5
C8—C7—H7A	108.9	C10—C12—H12C	109.5
C5—C7—H7B	108.9	H12A—C12—H12C	109.5
C8—C7—H7B	108.9	H12B—C12—H12C	109.5
H7A—C7—H7B	107.7		
			
O1—C1—C2—O2	2.23 (19)	C4—C5—C7—C8	93.95 (16)
C6—C1—C2—O2	−177.37 (13)	C6—C5—C7—C8	−85.35 (15)
O1—C1—C2—C3	−177.87 (13)	C5—C7—C8—O3	−74.53 (15)
C6—C1—C2—C3	2.5 (2)	C5—C7—C8—C9	160.84 (12)
O2—C2—C3—C4	178.56 (15)	C10—O5—C9—O4	3.3 (2)
C1—C2—C3—C4	−1.3 (2)	C10—O5—C9—C8	−174.98 (12)
C2—C3—C4—C5	−0.4 (2)	O3—C8—C9—O4	167.89 (15)
C3—C4—C5—C6	0.8 (2)	C7—C8—C9—O4	−71.30 (18)
C3—C4—C5—C7	−178.48 (14)	O3—C8—C9—O5	−13.79 (18)
O1—C1—C6—C5	178.33 (12)	C7—C8—C9—O5	107.02 (14)
C2—C1—C6—C5	−2.1 (2)	C9—O5—C10—C11	−156.25 (14)
C4—C5—C6—C1	0.41 (19)	C9—O5—C10—C12	81.06 (17)
C7—C5—C6—C1	179.74 (13)		

### Hydrogen-bond geometry (Å, °)

**Table:**

*D*—H···*A*	*D*—H	H···*A*	*D*···*A*	*D*—H···*A*
O1—H1···O4^i^	0.82	1.96	2.7621 (14)	164
O2—H2···O3^ii^	0.82	1.93	2.7417 (15)	169
O3—H3···O1^iii^	0.82	2.00	2.7832 (14)	160

Symmetry codes: (i) −*x*+1, −*y*+1, −*z*; (ii) −*x*+5/2, *y*+1/2, −*z*+1/2; (iii) −*x*+3/2, *y*−1/2, −*z*+1/2.
